# Special Issue: Cotton Molecular Genomics and Genetics 2.0

**DOI:** 10.3390/ijms252011255

**Published:** 2024-10-19

**Authors:** Shuaishuai Cheng, Qian-Hao Zhu, Jie Sun

**Affiliations:** 1 Key Laboratory of Oasis Eco-Agriculture, College of Agriculture, Shihezi University, Shihezi 832000, China; cheng_s_s@163.com; 2 CSIRO Agriculture and Food, GPO Box 1700, Canberra, ACT 2601, Australia

Cotton is a multiuse economic crop cultivated worldwide. Cotton fibers are the most important natural product of the textile industry, and cotton seeds are rich in oil and protein, providing high-quality oil for human consumption and feed for animals. Developing high yielding cotton varieties with not only superior fiber and seed quality but also strong resistance to biotic and abiotic stresses is the common goal of all cotton breeding programs. Understanding the molecular mechanisms regulating the biological processes contributing to the development of cotton fiber and seed as well as to stress resilience is essential for breeding the desired cotton varieties. Significant progress has been made in cotton molecular genetics, phenomics, proteomics, transcriptomics, metabolomics, and genomics [1] since the completion of the first Special Issue on the theme of “Cotton Molecular Genomics and Genetics”. The second Special Issue on the same theme was thus launched to capture the latest advancements, with a total of nine original research papers on various topics of cotton molecular genetics and genomics published.

Liu et al. [2] compared the fiber development processes of *Gossypium hirsutum* and *Gossypium barbadense* by means of transcriptomics and identified 42 candidate genes related to fiber elongation, with the roles of *GbAAR3* and *GbTWS1* in cell elongation being preliminarily verified in *Arabidopsis thaliana*. The authors also successfully developed single nucleotide polymorphism (SNP) markers for a total of 48 genes identified to contribute to the differential development of the secondary cell wall between *Gossypium hirsutum* and *Gossypium barbadense*, and demonstrated the potential role of the genes in regulating fiber strength and micronaire based on association analysis of a BC_3_F_5_ population using the gene-oriented SNPs. Because of the superior fiber quality of *Gossypium barbadense*, it has always been the goal of breeders to breed cotton varieties with a high yield, wide adaptability, and excellent fiber performance through interspecific hybridization with upland cotton [3,4]. The identification of genes associated with the development of superior fibers in *Gossypium barbadense* and development of SNP markers in these genes are the first steps towards accurately introgressing the superior fiber quality of *Gossypium barbadense* into upland cotton. In the long run, more comprehensively comparative analyses of *Gossypium barbadense* and *Gossypium hirsutum* fiber transcriptomes are necessary to identify genes contributing to various fiber traits and to develop molecular markers for molecular breeding of the traits.

Using a pink petal cotton mutant, Shao et al. [5] elucidated the reasons for the color formation of the mutant through comparative metabolomics and transcriptomics analyses. The authors demonstrated that anthocyanin-3-o-glucoside, paeoniflorin, and glycoside derivatives of paeoniflorin are the main pigments for the color change of the mutant’s pink petals, and identified *MYB21*, *UGT88F3*, *GSTF12*, and *VPS32.3* to be the key genes for anthocyanin biosynthesis and accumulation. Anthocyanin synthesis and accumulation are of great significance for fiber color [6], heterosis utilization [7], and improvement of reproductive capacity [8]. The results of the study provide new understandings for the biochemical and molecular mechanisms of anthocyanin biosynthesis and accumulation in upland cotton, and offer theoretical basis and germplasm resources for the utilization of anthocyanin.

The *Gossypium* genus includes more than 50 species, with most of them being wild species. Even within the cultivated cotton species, there are many primitive races (semi-wild) or landraces, which are good germplasm for mining genes contributing to stress resilience. In the study reported by Peng et al. [9], 45 accessions of the semi-wild cotton species (*Gossypium purpurascens*) were used to evaluate their salt-tolerance and to identify nine salt-tolerance candidate genes, including *MYB4* and *MYB105*, through transcriptomics analysis. The results demonstrated the potential importance of uncultivated cotton germplasm in broadening the genetic diversity of abiotic stress tolerance in the cultivated cotton species, which has depleted genetic diversity due to domestication and selective breeding; however, how the genes identified in semi-wild cotton species can be used in the cultivated cotton species is still waiting to be explored. On the same topic, Xue et al. [10] reported identification of the SBT family genes in the cultivated tetraploid species and their diploid ancestor species, given that some of them have been found to be involved in the salt-stress response in other plants. The authors identified 146, 138, 89, and 84 *SBT*s from *Gossypium hirsutum*, *Gossypium barbadense*, *Gossypium arboreum*, and *Gossypium raimondii*, respectively, which provide a basis for further exploring their role in salt tolerance, and offer candidate genes for genetic engineering and/or breeding of salt–alkali tolerance once the function of the candidate genes has been experimentally characterized.

Chalcone isomerase (CHI) is one of the most important enzymes of the pathway involved in the biosynthesis of flavonoids, which have a wide range of biological functions in plant growth and development, including stress tolerance. In the study carried out by Zu et al. [11], the four homologous *CHI* genes were characterized in terms of their role in resistance to Fusarium wilt, based on an investigation of their expression levels and subcellular localization and the content of the corresponding metabolites and endogenous hormones as well as reactive oxygen species. It was indicated that the four homologous *CHI* genes might play a synergistic role in resistance to Fusarium wilt. 

Transgenic cotton varieties containing insect-resistant gene(s) from *Bacillus thuringiensis* (Bt) have been widely adopted in cotton production. Preventing insects from developing resistance to Bt genes is vital for the sustainability of transgenic Bt cotton and the cotton industry worldwide. Dhammi et al. [12] observed that an increasing temperature would raise the feeding rates of tobacco budworm (*Chloridea virescens*) and cotton bollworm (*Helicoverpa zea*), and an increased feeding rate was even observed for tobacco budworm on extracts from Bollgard II plants containing two insecticide Bt proteins (Cry1Ac and Cry2Ab2), consequently reducing the susceptibility and mortality of the insects to Bt proteins. While this observation requires confirmation by further experiments, the phenomenon, if confirmed and widespread, suggests that more stringent refuge measures need to be implemented for Bt crops under the conditions of global warming.

Cotton seed is a major source of oil for human consumption and the oil content of cotton seeds is also important for seed germination and seedling establishment. The work by Zhong et al. [13] reported the identification of the long-chain acyl-coenzyme A synthetase (LACS) gene family related to lipid biosynthesis in four cotton species, including the two cultivated tetraploid cotton species and their diploid progenitors. The work was a routine gene family analysis but one interesting finding was that almost all *LACS* genes were found to be have a higher expression level in high seed oil content variety than in low seed oil content variety. At present, while many works have been carried out regarding cottonseed quality, the development of cotton varieties with a high seed quality, yield, and fiber quality is far from ideal due to the negative correlation among the yielding traits and seed and fiber quality traits [14]. 

Lint percentage is one the most important yield components. Because of its importance, numerous studies, including QTL (quantitative trait locus) mapping and GWAS (genome-wide association analysis), have been carried out, aiming for identification of the major genetic loci regulating the trait. The study by Wang et al. [15] used the GWAS to investigate genetic loci associated with lint percentage, but the materials used in the study were mainly upland cotton races (a total of 188) rather than normal varieties of the species. The authors identified a total of 274 SNPs to be significantly associated with lint percentage, with 11 of them being identified to be associated with lint percentage in two or more environments. Further investigating the expression and annotation of the genes in the SNP regions led the authors to propose *Gh_D12G0934* and *Gh_A08G0526* as the candidate genes for fiber initiation, elongation and lint percentage. While the materials used in the study were unique compared to other similar studies, they were genotyped by using the CottonSNP80K chip, which was designed based on SNPs identified in upland cotton; therefore, the unique loci associated with lint percentage in the upland cotton races might have been missed out. If genome sequencing-based approaches were used in genotyping the materials and SNP identification, the study would have provided more insightful outcomes.

In cotton, *Agrobacterium*-mediated genetic transformation is the most widely used method for integrating exogenous genes. However, somatic embryogenesis in cotton is highly genotype-dependent, so only a few genotypes can be used as the transforming explant tissues, severely limiting cotton genetic engineering. Currently, it is very difficult to genetically transform most cotton varieties with excellent agronomic traits, and the regeneration and transformation systems currently established in the transformable accessions are inefficient and time-consuming [16,17,18]. Genetic improvement of the regeneration ability of cotton is a key strategy to overcome the difficulties in cotton genetic transformation [17]. The expression of *WUSCHEL* (*WUS*), the earliest gene identified in the *WOX* (*WUSCHEL-related homeobox*) gene family, is an early hallmark of acquisition of shoot apical meristem (SAM) and de novo establishment of SAMs is critical for plant regeneration. Sun et al. [19] carried out a genome-wide identification of the *WOX* family genes and identified 40 *WOX* genes in the upland cotton genome. Based on RNA-seq, the authors found that *GhWOX4* and *GhWOX13* might play roles in almost all stages of somatic embryogenesis, while *GhWOX2* and *GhWOX9* might play roles in the later stages of embryo shaping and embryo development. A co-expression analysis was carried out and revealed genes involved in a wide range of biological processes being co-expressed with *WOX* genes, indicating the complexity of the *WOX* gene network related to somatic embryogenesis. Although the roles of the proposed genes in somatic embryogenesis have not been functionally characterized, the results provide useful information for the potential functions of *WOX* genes during cotton somatic embryogenesis and potential candidate genes for investigating their specific regulatory roles in somatic embryogenesis.

Genomics studies are essential for understanding gene families, genetic diversity, and genetic variations and their association with agronomic traits of interest. Genomics studies also provide tools for molecular breeding. It is thus not surprising that this Special Issue attracted the interest of the cotton community. We hope that the articles published in this Special Issue provide useful information to further explore the related topics and to finally generate impactful results for genomics-enabled cotton breeding.
